# The effect of anxiety, depression, and structural social capital on life satisfaction among people with hearing disabilities: a cross-sectional study in Shanghai, China

**DOI:** 10.3389/fpsyt.2023.1164324

**Published:** 2023-10-05

**Authors:** Xiaomin Wei, Ting Wang, Yuxin Zhang, Nan Jiang, Quqing Wang, He Cao, Xinrui Shi, Jiwei Wang

**Affiliations:** ^1^Shanghai Municipal Center for Health Promotion, Shanghai, China; ^2^Key Lab of Health Technology Assessment of Ministry of Health, School of Public Health, Fudan University, Shanghai, China

**Keywords:** life satisfaction, anxiety, depression, structural social capital, people with hearing disabilities

## Abstract

**Background:**

Life satisfaction (LS) serves as a crucial indicator of social wellbeing and plays a significant role in formulating strategies aimed at enhancing health outcomes among the hearing-disabled population. This study aimed to examine the effect of anxiety, depression, and structural social capital on life satisfaction among people with hearing disabilities in Shanghai, China.

**Methods:**

A cross-sectional study was conducted in Shanghai, China. As of March 2022, 337 people with hearing disabilities were recruited from the Shanghai Disabled Persons' Federation. An online survey was conducted using a four-part questionnaire to collect data including demographic characteristics, the Hospital Anxiety and Depression Scale (HADS), the Social Capital Scale (SCS), and a single-item question to measure life satisfaction. One-sample *t*-tests, Pearson's correlation analysis, and hierarchical multiple regression analysis were performed.

**Results:**

Anxiety (β = – 0.153) and depression (β = – 0.242) were significant factors influencing life satisfaction among people with hearing disabilities. Structural social capital also played an influential role in life satisfaction, and people with hearing disabilities who lack social networks (β = 0.125) and social support (β = 0.121) reported significantly lower levels of life satisfaction. However, no significant relationship was found in this study between LS and other components of structural social capital, such as social participation.

**Conclusion:**

This study shows that paying attention to mental health is critical for people with hearing disabilities to achieve social wellbeing and promote LS improvement. At the same time, the government and society also need to focus on the structural social capital, provide various social service programs, enhance social support, and expand social networks, improving LS for people with hearing disabilities.

## 1. Introduction

Hearing disability is an invisible health condition with significant implications for an individual's life satisfaction (LS) (1). The impact of hearing disabilities may be profound, with consequences for social, functional, and psychological wellbeing, which in turn affect the life satisfaction of the person (2). Studies have found that people with hearing disabilities have lower overall LS compared to people with normal hearing (3).

LS is defined as a cognitive perception and overall evaluation of the quality of life of an individual as a whole, forming an integral component of their subjective sense of wellbeing (4). LS does not differentiate individuals based on physical abilities or disabilities and can be accurately measured, irrespective of an individual's physical disability (5). At the same time, measuring LS is also an essential indicator of social wellbeing, and it is increasingly advocated to be applied in public policy (6). Previous studies have introduced several tools designed to assess LS levels in populations, including the Satisfaction with Life Scale (4), the Life Satisfaction Index (7), and the Riverside Life Satisfaction Scale (8). The single-item measure is another commonly used method for assessing LS (9). These measures employ a single statement or question to generate an overall score of LS, and the results appear to align with those obtained from lengthier scales and inventories. Researchers compared a single measure of LS with the Satisfaction with Life Scale and observed no substantial differences between the two measures (10).

LS is influenced by a range of factors, such as sociodemographic variables, economic income, educational attainment, physical health status, mental wellbeing, social networks, and social support (11–13). Existing research indicates that determinants of LS among the hearing disability population are associated with the degree of hearing disabilities, daily life consequences of hearing disabilities, limited social interactions, and mental wellbeing (2, 14). It is also well known that mental disorders such as depressive or anxiety affect LS negatively (15, 16). People with hearing disability experience heightened negative emotions and are more prone to developing adverse mental conditions such as anxiety and depression, which may harm their LS (17, 18). However, contradictory findings have been reported in previous studies. It has been often replicated that severe health issues are not necessarily associated with low LS (19). Individuals usually rate their living and health conditions as being better than one would assume, given their objective status (20). This phenomenon is commonly known as the “wellbeing paradox” (20). Investigators using a sign-language-based interview in Sweden noted that deaf older people had higher rates of depression than hearing individuals but that LS did not differ (21). The associations between anxiety and depression with LS are still unclear due to very limited empirical data in the literature.

Social capital was found to be a crucial factor related to LS (22, 23). While definitions of social capital vary, the commonly accepted concept is the one proposed by Putnam (24), which refers to “features of social organization, such as trust, norms, and networks, that can improve the efficiency of society by facilitating coordinated actions.” In general, social capital is frequently divided into cognitive and structural domains based on differences in their health effects (24, 25). The former refers to subjective aspects encompassing trust, reciprocity, and a sense of belonging, whereas the latter pertains to objective aspects including social networks, social support, and social participation (25). People with hearing disabilities often face communication difficulties due to hearing loss, which can restrict their engagement in social activities and hinder the maintenance of social networks (26). As a result, people with hearing disabilities have more restricted access to structural social capital. Meanwhile, structural social capital is tangible and can be readily observed by the existence of network ties as well as roles, rules, and procedures (27). In comparison with cognitive social capital, proposing feasible intervention programs for structural social capital is more straightforward (27). Therefore, it is essential to examine the impact of structural social capital on LS among individuals with hearing disabilities.

A better understanding of how mental health and structural social capital affect the LS of people with hearing disabilities is critical for developing interventions and policies to achieve social wellbeing. Therefore, the study aimed to examine the relationship between anxiety, depression, and structural social capital with LS among people with hearing disabilities in Shanghai.

## 2. Materials and methods

### 2.1. Study design and population

All participants in this study were registered members of the Shanghai Disabled Persons' Federation, and each of them possessed Chinese-certified hearing disability certificates. Associations of the deaf in seven districts of Shanghai (Huangpu, Pudong, Changning, Putuo, Hongkou, Baoshan, and Jingan) were willing to participate in this research. Each district's association of the deaf has a WeChat group that includes all certified members. There are ~150–200 people with hearing disabilities in each district's WeChat group, and all of them are informed through the WeChat group to voluntarily participate in the questionnaire survey. Before the start of the study, a pre-experimental questionnaire was administered to people with hearing disabilities. A total of eight people with hearing disabilities participated in the pre-survey accompanied by sign language interpreters and experts. The study aimed to determine the level of comprehension of the questionnaire content by people with hearing disabilities and to develop an experimental version of the questionnaire. In the formal study, the survey method was an online questionnaire, but the study still retained the online communication channel and WeChat official account so that experts can answer the relevant questions of the participants. In this study, the sample size was calculated based on an estimate of the prevalence of anxiety reported by 31.3% of people with hearing disabilities (28). Under the absolute error of 4%, considering the underestimation of the prevalence of anxiety and depression in previous studies, it was estimated that at least 263 people with hearing disabilities should be included in the study. In this study, the inclusion criteria were as follows: (1) individuals with hearing disabilities holding disability certificates certified by the China Disabled Persons' Federation, which primarily encompassed permanent hearing disabilities of varying degrees in both ears caused by different reasons, resulting in an inability to hear or perceive surrounding sounds; (2) age between 18 and 70 years; and (3) possessing a basic ability to read and comprehend. As of 1 March 2022, we received a total of 357 completed questionnaires. Of the participants, 20 did not pass a logical quality test. The current analysis was based on data from 337 (94.4%). All participants were informed that they were providing data for a scientific study, all of which were confidential and anonymous, and would be used only for the study. Participant consent was obtained along with their responses to the questionnaire. Approval for the research was received from the Ethics Committee of the Public Health School of Fudan University.

### 2.2. Measurement

#### 2.2.1. Sociodemographic characteristics

Data on age, gender, marital status, educational level, working status, and the type of hearing disabilities were collected using a self-reported questionnaire.

#### 2.2.2. Life satisfaction

Overall LS was assessed using a single-item on a 5-point Likert scale (ranging from 1 = not satisfied at all to 5 = very satisfied). Cheung and Lucas (10) reported that single-item LS measures exhibited comparable performance to other multiple LS instruments.

#### 2.2.3. Anxiety and depression

Symptoms of anxiety and depression as measured by means of the hospital anxiety and depression scale (HADS) (29) in its validated Chinese version (30). The HADS is a 14-item scale that measures the presence and severity of symptoms of anxiety and depression over the past week. It combines a seven-item subscale for anxiety (HADS Anxiety) and a seven-item subscale for depression (HADS Depression), both omitting somatic symptoms, to minimize false positives due to medical illness. Overall, it has demonstrated satisfactory psychometric properties in different groups: primary care patients (31), cancer inpatients (32), physically disabled people (33), and general populations (31, 34). Previous studies have demonstrated that the HADS scale can be recommended for assessing psychological distress in the general population aged 65–80 years, with acceptable internal consistency (35). In addition, research confirmed that the HADS also has good psychometric properties in community samples (34). Items are scored on a 0–3 Likert scale, with a 0 to 21 global score range for both anxiety and depression, with scores from 8 to upward generally considered positive for symptomatology of increasing severity. Previous studies have shown high internal consistency and evidence of structural validity for the scale (31).

#### 2.2.4. Social capital

Social capital was measured by the scale which was developed by the research group (36). The three dimensions of structural social capital in this scale include social participation, social network, and social support. Social participation gauges participants' engagement in activities such as volunteer work, social group gatherings, community events, or leisure activities. Social network measures the closeness of participants' relationships with friends, relatives, and others, emphasizing the strength of their connections. Social support assesses whether participants can receive help from others when needed and whether anyone is willing to lend a listening ear. The scale's timeframe covers 1 month, and all items are standardized on a 5-point Likert scale, ranging from 1 (never) to 5 (always). Higher summary scores indicate higher levels of social capital. Cronbach's alpha coefficient value is 0.933.

### 2.3. Statistical analyses

All statistical analyses were performed using IBM SPSS Statistics 22.0 software (IBM Corp. Armonk, NY, USA). Categorical variables were described using frequencies and percentages, while continuous variables were described using means and standard deviations. One-sample *t*-tests were conducted to compare the current mean anxiety and depression scores, structural social capital scores, and LS scores to means reported in previous studies. The relationship between anxiety, depression, structural social capital, and LS was analyzed using the Pearson correlation coefficient. A hierarchical multiple regression analysis was conducted to verify the relationships between sociodemographic variables, anxiety, depression, structural social capital, and LS among people with hearing disabilities and to identify factors with high explanatory power related to LS. Variance inflation factor (VIF) and tolerance tests assessed the multicollinearity of variables. Two-tailed statistical significance level was set at 0.05.

## 3. Results

### 3.1. Participants' characteristics

Table 1 summarizes the demographic characteristics of the participants. In the study, 337 people with hearing disabilities were recruited, of which 159 were aged 60 or less (47.2%) and 132 were men (39.2%). A great majority of them were married (80.4%) and retired (76.0%). Only 46.8% of people had received high school, technical secondary school, and above education.

**Table 1:** Demographic characteristics of the respondents.

**Variables**		***N* (%) (*n =* 337)**
Gender	Male	132 (39.2)
	Female	122 (60.8)
Age (years)	≤ 60	159 (47.2)
	>60	178 (52.8)
Marital status	Married	271 (80.4)
	Unmarried	66 (19.6)
Educational level	Elementary school and below	40 (11.9)
	Primary school, high school, and secondary school	276 (81.9)
	Bachelor's degree or above	21 (6.2)
Working status	On-the-job	81 (24.0)
	Retire	256 (76.0)

### 3.2. Mental health scores compared to normative studies

Table 2 presents descriptive data on anxiety and depression from the current study, as well as data from previous studies using the same HADS scale. The HADS has been found to be a valid scale for assessing the severity and extent of anxiety and depressive symptoms in somatic, psychiatric, and primary care patients, as well as in the general population, with sub-scores of >7 in the corresponding subset being a clear indicator of anxiety or depression. The mean scores of the HADS-A and HADS-D in this study were 7.9 (SD = 3.3) and 9.6 (SD = 3.5), respectively, which fall in the “normal” range of indicators of anxiety and depression according to the recommended guidelines. The mean scores of both anxiety and depression scores were significantly higher in the hearing disability population than that of the clinical sample (*t* = 33.50, *P* < 0.001; *t* = 54.75, *P* < 0.001). In addition, in the general population, the mean scores in the current study were also significantly higher than the scores in Wong and Fielding (38) study of the non-clinical Hong Kong general adult population (*t* = 24.54, *P* < 0.001; *t* = 32.86, *P* < 0.001) and the scores of Hinz et al. (40) on the Colombian general adult population sample (*t* = 15.43, *P* < 0.001; *t* = 22.95, *P* < 0.001).

**Table 2:** HADS descriptive statistics for the present and normative studies.

**Study and sample**	***n***	**HADS-A**		**Comparison to the present study**		**HADS-D**		**Comparison to the present study**	
		**Mean**	**SD**	***t***	***p***	**Mean**	**SD**	***t***	***p***
Present study	337	7.9	3.3	-	-	9.6	3.5	-	-
Internal medicine outpatients at the general hospital in Shanghai, China (37)	6,172	3.3	2.4	33.50	<0.001	2.6	2.2	54.75	<0.001
General population over 18 years old in Hong Kong, China (38)	5,001	3.6	3.1	24.54	<0.001	3.3	3.4	32.86	<0.001
Patients presenting to clinics of general hospitals in Guangzhou, China (39)	2,408	3.4	2.9	26.21	<0.001	3.8	3.3	29.99	<0.001
Adults in the Colombian general population (18 and over) (40)	1,500	4.6	3.6	15.43	<0.001	4.3	3.9	22.95	<0.001

### 3.3. Structural social capital scores compared to normative studies

Table 3 presents descriptive data for structural social capital in the current study, as well as from previous studies using the same scale. The mean scores for each dimension of structural social capital (including social networks, social participation, and social support) for the hearing disability population were significantly lower than the previous social capital scores of Chen et al. (41) study on a general population sample in Shanghai (*t*^*a*^= −14.80, *p*^*a*^ < 0.001; *t*^*b*^= −11.60, *p* ^*b*^ < 0.001; *t*^*c*^= −3.13, *p*^*c*^= 0.002) and Fang et al. (42) on breast cancer survivors (*t*^*a*^= −9.83, *p*^*a*^ < 0.001; *t*^*b*^= −19.86, *p*^*b*^ < 0.001; *t* = −15.52, *p*^*c*^ < 0.001).

**Table 3:** Structural social capital descriptive statistics for the present and previous studies.

**Study and sample**	***n***	**Mean (SD) -Structural social capital**			**Comparison to present study**					
		**Social networks**	**Social participation**	**Social support**	***t^*a*^***	***p^*a*^***	***t^*b*^***	***p^*b*^***	***t^*c*^***	***p^*c*^***
Present study	337	2.93 (0.84)	2.58 (0.81)	2.99 (0.73)	-	-	-	-	-	-
A sample of 600 people aged 15-69 years in Shanghai, China (41)	640	3.66 (0.67)	3.08 (0.53)	3.12 (0.55)	−14.80	<0.001	−11.60	<0.001	−3.13	0.002
532 Breast Cancer Survivors in Shanghai, China (42)	532	3.48 (0.78)	3.65 (0.75)	3.69 (0.59)	−9.83	<0.001	−19.86	<0.001	−15.52	<0.001

t^a^ and p^a^ represent the social networks, t^b^ and p^b^ represent the social participation, t^c^ and p^c^ represent the social support.

### 3.4. Life satisfaction scores compared to normative studies

Table 4 lists descriptive data on LS in the current study, as well as data from other studies that used single-entry LS questions. The mean LS scores in the current study were significantly lower than the LS scores of Yuan (43) for the adult cohort sample in Shanghai and elsewhere (*t* = −2.15, *p* = 0.032), Seo et al. (44) for a nationally representative sample in China (*t* = −5.26, *P* < 0.001), and Cheung and Lucas (10) for a large population sample in Washington (*t* = −2.62, *p* = 0.009).

**Table 4:** LS descriptive statistics for the present and normative studies.

**Study and sample**	***n***	**single-item life satisfaction (range 1–5)**		**Comparison to the present study**	
		**Mean**	**SD**	***t***	***p***
Present study	337	3.31	0.72	-	-
A random sample of adults from three regions of China: Beijing, Shanghai, and Guangdong Province (43)	6,002	3.43	1.01	−2.15	0.032
A nationwide population sample from the 2016 China Family Panel Studies (CFPS) survey (44)	31,368	3.62	1.08	−5.26	<0.001
large population sample in Washington from the 2010 BRFSS (10)	13,064	3.40	0.62	−2.62	0.009

### 3.5. Relationship between anxiety, depression, structural social capital, and life satisfaction

Table 5 presents the Pearson correlation coefficients of the relationship between anxiety, depression, structural social capital (social participation, social network, social support), and LS. The results indicated anxiety, depression, and structural social capital as significantly correlated with the LS. The internal consistency reliability of anxiety and depression estimates for this study was 0.808 and 0.764, respectively. In this study, the social capital scale demonstrated good internal consistency reliability, with a Cronbach's α value of 0.833. Additionally, the component scales of social participation, social network, and social support exhibited high internal consistency, with Cronbach's α values of 0.913, 0.867, and 0.860, respectively.

**Table 5:** Pearson's correlation coefficients values regarding study variables.

**Characteristic variable**	**1**	**2**	**3**	**4**	**5**	**6**
1. Anxiety (Cronbach's α 0.808)	-					
2. Depression (Cronbach's α 0.764)	0.377^**^	-				
3. Social participation (Cronbach's α 0.913)	0.043	−0.119^*^	-			
4. Social network (Cronbach's α 0.867)	−0.215^**^	−0.290^**^	0.484^**^	-		
5. Social support (Cronbach's α 0.860)	0.115^*^	−0.048	0.465^**^	0.294^**^	-	
6. Life satisfaction	−0.261^**^	−0.352^**^	0.238^**^	0.314^**^	0.198^**^	-

^*^P < 0.05, ^**^P < 0.01.

### 3.6. Factors related to life satisfaction

Table 6 shows the unstandardized (B) and standardized (β) regression coefficients for model 1, model 2, and model 3. In model 1, it was found that education level (β = 0.120, *P* = 0.039) was correlated with LS among hearing-disabled patients. The model improved significantly when anxiety and depression were included (Model 2) explaining 15.0% of the variance of LS (*P* < 0.001). In model 2, anxiety (β = – 0.137, P = 0.010) and depression (β = – 0.300, *P* < 0.001) were significantly related to LS. For model 3, social capital variables including social participation, social network, and social support were entered into the model. The model improved significantly when social capital variables were included (Model 3) explaining 21.6% of the variance of LS (*P* < 0.001). Anxiety (β = – 0.153, P = 0.004) and depression (β = −0.242, *P* < 0.001) were still significant factors of LS, and social network (β = 0.125, *P* = 0.029) and social support (β = 0.121, *P* = 0.026) explained a significant amount of variance of LS, while social participation (β = 0.097, P = 0.096) did not show a significant relationship with LS.

**Table 6:** Summary of hierarchical regression analysis for life satisfaction.

	**Model 1**			**Model 2**			**Model 3**		
	***B***	**SE** ***B***	β	***B***	**SE** ***B***	β	***B***	**SE** ***B***	β
Gender	0.023	0.077	0.016	0.022	0.071	0.015	0.037	0.069	0.026
Age	0.065	0.093	0.046	0.064	0.086	0.045	0.011	0.085	0.008
Marital status	−0.047	0.094	−0.026	−0.051	0.087	−0.029	−0.007	0.084	−0.004
Educational level	0.110	0.053	0.120^*^	0.071	0.051	0.077	0.043	0.049	0.047
Work status	0.070	0.116	0.041	0.041	0.108	0.024	0.026	0.104	0.015
Anxiety				−0.195	0.076	−0.137^**^	−0.218	0.075	−0.153^**^
Depression				−0.437	0.076	−0.300^***^	−0.353	0.076	−0.242^***^
Social participation							0.017	0.010	0.097
Social network							0.023	0.011	0.125^**^
Social support							0.022	0.010	0.121^*^
R^2^	0.012			0.154			0.220		
R^2^ change	-			0.142			0.066		
F	0.967			9.280^***^			10.040^***^		

^*^P < 0.05, ^**^P < 0.01, and ^***^P < 0.001; B, unstandardized regression coefficient; SE, standard error of unstandardized regression coefficient; β, standardized regression coefficient.

## 4. Discussion

In this study, people with hearing disabilities in Shanghai compared with the other population exhibit significantly higher levels of anxiety and depression, lower levels of social capital, and lower levels of LS. Among these, anxiety and depression were significant factors affecting LS in people with hearing disabilities, and lack of social networks and social support was associated with lower levels of LS.

The findings strongly support early discoveries concerning psychological distress in the deaf population. The Norwegian Postal Service conducted two separate surveys: one assessing symptoms of depression and anxiety in the general population and the other in the deaf population (45). The results indicated that the deaf population exhibited higher levels of psychological distress compared to the general population (45). These differences may be attributed to factors such as the availability and frequency of medical interventions, disparities in deaf education opportunities, societal attitudes, and everyday communication challenges (17, 46, 47). Additionally, the low levels of structural social capital may be due to communication barriers and other issues related to hearing disabilities that restrict their ability to establish and expand social relationships, making it more challenging for them to develop and maintain friendships and networks compared to the general hearing population, ultimately resulting in decreased availability or even a deficit of social capital (48–50). There may be several explanations for the low levels of LS found in the present study. Communication challenges associated with hearing disabilities may intensify feelings of isolation and negatively impact subjective wellbeing. The presence of high-risk mental health issues and experiences of social discrimination among this population are also potential factors that can diminish their LS (21, 51). Regarding the wellbeing paradox, our findings somehow contradict this phenomenon to some extent. The hearing disability population did report lower life satisfaction compared to the general population, but this finding should be interpreted with caution. This study was compared to previously published works; however, the demographic characteristics of our sample did not match those of previous studies. Without direct intergroup comparisons, it is impossible to determine the exact differences in anxiety, depression, structural social capital, and LS between the hearing disability population and other groups. However, the current results provide evidence that the hearing disability population may be experiencing psychological distress, and their quality of life may be suboptimal. This further highlights the insufficient attention and support provided to the population by the Shanghai municipal government and relevant institutions.

Higher educational attainment is associated with employment opportunities, a sense of achievement in pursuing goals, and improved social status, all of which can promote self-esteem, buffer the impact of stress, and increase LS (52). For people with hearing disabilities, they have limited access to higher education (53). However, education plays an increasingly vital role in the rapid development of science and technology as a means of improving their living conditions and occupational status (54). On the one hand, studies have found that educational attainment is a key determinant of employment status and income levels, particularly for people with hearing disabilities (55). Due to their low educational qualifications, individuals with hearing disabilities are often engaged in manual labor with low skill requirements and limited alternatives (56). It leads to considerably inferior job stability, lower income levels, and poor working conditions, all of which have a negative impact on the LS among people with hearing disabilities. On the other hand, education can serve as a compensatory mechanism for overcoming the communication barriers caused by hearing disabilities, thereby broadening their social opportunities and employment prospects (57).

Anxiety and depression, common psychological problems in people with hearing disabilities (58), play a crucial role in the development of stress related to modern life for people with hearing disabilities and influence the evaluation of LS in multiple ways (45). Individuals with hearing disabilities who experience high levels of anxiety and depression often struggle with low self-esteem and lack of confidence, which are frequently attributed to the stigma surrounding hearing disabilities (59). They generally have a tendency to judge themselves negatively and are often unable to enjoy life as a normal person because they feel worthless (46). Additionally, low levels of self-esteem and poor self-image can contribute to significant stress and distress, directly or indirectly impacting the LS of individuals with hearing disabilities (46). Furthermore, feelings of loneliness as a result of experiencing social isolation and discrimination are regularly observed among people with hearing disabilities (60), and to some extent, anxiety and depression can exacerbate feelings of loneliness in people with hearing disabilities, further reducing their LS (61). Therefore, timely attention to anxiety and depression of hearing-disabled people and early implementation of interventions to reduce the occurrence of adverse psychological effects can help improve the LS of people with hearing disabilities.

Previous research has shown that social network is an influential factor in LS in people with hearing disabilities (62). For people with hearing disabilities, when language and self-efficacy resources are reduced, social capital can compensate for the lack of other resources (50). On the one hand, individuals with hearing disabilities can mitigate the communication barriers and distress resulting from their physical abilities by actively engaging in social networks and activities, thereby enhancing the value of their limited social connections (63). Moreover, members of the hearing-disability groups have a natural closeness to each other and form natural attachments and a strong sense of identity, which subsequently internalize the psychological and behavioral characteristics of hearing-disabled groups to cope with the difficulties and obstacles that arise in their lives, helping to reduce the occurrence of undesirable problems and increase the LS of hearing-disabled groups (64). On the other hand, the larger the size of the social network of people with hearing disabilities, the larger the number of social relationships they have, indicating more diverse social support (65). When people with hearing disabilities face a crisis, they are more likely to seek help, thereby increasing LS in the population. Additionally, research has demonstrated that larger social networks enable individuals to expand their exposure to the external world, fostering greater experiences of positive emotions and decreased perception of negative emotions, ultimately enhancing their LS (66). Furthermore, establishing a social network of adequate size offers individuals with hearing disability additional avenues for recreation and communication, alleviating feelings of loneliness and anxiety and consequently enhancing their LS (67).

Research has shown that higher levels of social support can effectively enhance LS by fostering self-esteem, increasing belonging, and relieving life stress (68). Regarding self-esteem, individuals experience an enhanced sense of self-esteem when they receive greater support from friends, family, and others, enabling them to pursue their aspirations, satisfy their needs for autonomy and pleasure, and consequently elevate their LS (69). Sufficient social support not only offers physical and mental assistance but also enhances individuals' adaptability to their environment, effectively mitigating feelings of crisis and societal stigma and fulfilling their needs (70). In addition, people with hearing disabilities can enjoy a better life with reliable emotional support in an active community. Emotional social support plays a crucial role in alleviating psychological stress, improving the physical and mental wellbeing of individuals with hearing disabilities, and enhancing their LS (71). Therefore, it is essential for society to prioritize the needs of individuals with hearing disabilities, ensuring the provision of a user-friendly environment, comprehensive support, and continuous expansion of social support mechanisms to enhance their LS.

Taking into consideration the relatively lower levels of mental health, social capital, and LS among people with hearing disabilities and the significant impact of their mental health and structural social capital on LS, it is crucial to prioritize interventions aimed at improving factors such as anxiety and depression symptoms, social networks, and social support among this population, whose efforts can have a positive impact on their overall LS. It is recommended that the government and other institutions proactively provide mental health support and interventions. Additionally, efforts should be made to facilitate the accumulation of support resources, such as social capital, to enhance their sense of wellbeing.

There are still limitations in this study, mainly in the following aspects: First of all, this study used convenient sampling to recruit hearing-disabled patients, so the sample may not be sufficiently representative. Second, this study used an online survey published through WeChat. On the one hand, hearing loss appeared to negatively impact cognitive function, and we could not confirm the accuracy of the data provided by the participants. On the other hand, due to the limitations of the Internet survey, we are unable to explain the questions face to face. Therefore, the subjects included were hearing-disabled persons with basic literacy skills to reduce misinterpretation of the questions by the participants in the study. However, this might have resulted in selection bias among the study participants. Furthermore, employing an online questionnaire raises concerns about potential behaviors such as hasty completion without thorough reading, which could compromise the study's quality. To address this issue, logic tests were incorporated into the questionnaire to deter individuals from completing it without carefully reading the questions. Future research should consider the use of other forms of data collection, such as personal interviews with sign language interpreters. Third, our study methodology used a cross-sectional study design, which has the disadvantage of insufficient evidence of causal relationships between the study variables to conclude the direction of the effects. Future longitudinal studies are needed to elucidate the direction.

## 5. Conclusion

Anxiety, depression, social networks, and social support are closely associated with the LS of people with hearing disability. Mental wellbeing and social capital should be prioritized as key factors for enhancing the subjective wellbeing of this population.

## Data availability statement

The original contributions presented in the study are included in the article/supplementary material, further inquiries can be directed to the corresponding authors.

## Ethics statement

The studies involving humans were approved by the Ethics Committee of the Public Health School of Fudan University. The studies were conducted in accordance with the local legislation and institutional requirements. The participants provided their written informed consent to participate in this study.

## Author contributions

TW and XW were responsible for the acquisition, analysis and interpretation of data, and the drafting of the manuscript. NJ and YZ contributed to the acquisition, interpretation of data, and critically reviewed the manuscript for important intellectual content. QW, HC, and XS participated in data interpretation and revised the manuscript. JW and XW was the project coordinator, contributed to the design of the study, reviewed, and revised the manuscript. All authors have read and approved the final manuscript.
